# Is Resistance Training to Muscular Failure Necessary?

**DOI:** 10.3389/fphys.2016.00010

**Published:** 2016-01-29

**Authors:** Sanmy R. Nóbrega, Cleiton A. Libardi

**Affiliations:** Laboratory of Neuromuscular Adaptations to Resistance Training, Department of Physical Education, Federal University of São CarlosSão Carlos, Brazil

**Keywords:** weight training, hypertrophy, voluntary fatigue, electromyography, concentric failure

## Introduction

Resistance training (RT) is the main method of exercise for improving strength and skeletal muscle mass (i.e., muscle hypertrophy; ACSM, 2009). In order to promote such adaptations, high-intensity resistance training (HI-RT) with loads above 60% of one repetition maximum (1-RM) are typically recommended (ACSM, 2009). To further maximize increases in strength and muscle hypertrophy, it has been suggested repetitions to muscular failure (Jacobson, 1981; Rooney et al., 1994; Schott et al., 1995; Drinkwater et al., 2005), which can be defined as the inability to move a specific load beyond a critical joint angle (i.e., sticking point; Drinkwater et al., 2005) or as incapacity to complete a repetition in a full range of motion due to fatigue (Izquierdo et al., 2006).

Some studies suggest that HI-RT to muscular failure promotes greater activation of motor units (MUs) compared to no failure HI-RT (Willardson, 2007; Akima and Saito, 2013). During a HI-RT session, MUs recruitment pattern follows the size principle, in which the low threshold MUs are recruited first, followed by high threshold MUs (Henneman, 1957). It has been speculated that even more high excitability threshold MUs, composed predominantly of type IIx muscle fibers, are recruited when repetitions are performed to failure, possibly due to fatigue in MUs (Willardson, 2007). In fact, RT to failure might promote increased electromyography (EMG) activity, which suggests increased recruitment of high threshold MUs (Akima and Saito, 2013), even when RT is performed at low intensities (Pincivero et al., 2006). In this regard, it is believed that recruiting as many MUs as possible results in maximal gains in muscle hypertrophy and strength on the target muscles (Wernbom et al., 2007). Despite a logical rationale, it is unclear if RT to failure is really necessary. Few studies directly compared RT to failure and no failure on muscle activation and strength. Results of these studies are conflicting, with some studies finding superiority for RT to failure and others showing no significant differences (Drinkwater et al., 2005; Izquierdo et al., 2006; Looney et al., 2015). Additionally, none of these studies assessed muscle hypertrophy.

Based on the current literature, it is still unclear if RT to muscular failure is really necessary to maximize increases in muscle strength and hypertrophy compared to no repetition failure. Thus, the purpose of this manuscript is to discuss the effects of RT to failure on MUs recruitment and adaptive responses (i.e., increases in strength and muscle mass), providing rationale as to why RT to failure might differently affect muscle adaptations in different populations.

## Muscular failure in high intensity resistance training

Few studies have attempted to directly evaluate the effects of HI-RT to muscle failure or no failure in neuromuscular adaptations (e.g., strength and muscle mass). Izquierdo et al. (2006) randomized 42 basque pelota players in two groups: (1) repetition failure (3 sets of 10-RM); (2) no repetition failure (~6 sets of 3–5 repetitions), with the same intensity (75% 1-RM) and volume. Results showed similar increases in muscular strength between training groups, independently of muscle failure. Neither fatigue levels nor muscle activation were assessed, leading us to speculate that the higher number of sets and fewer repetitions performed by the no repetition failure group resulted in a substantial level of fatigue. These findings suggest that, since subjects were untrained in strength, the resulting fatigue promoted maximal MUs recruitment previous to muscular failure point, boosting strength gains. Accordingly, Sundstrup et al. (2012), evaluated muscle activation of neck and shoulder muscles with EMG during lateral raise to failure with elastic tubing of different resistances in untrained women. Subjects performed a heavy load set (3-RM) and a repetitions failure set with lower resistance (~15-RM). Results indicated that the normalized EMG activity during the failure set was significantly lower in the first repetitions and significantly higher in the latter repetitions when compared to the 3-RM set, reaching an activation plateau 3–5 repetitions before concentric failure. It suggests that, for individuals untrained in strength, HI-RT to complete failure is not necessary for maximum recruitment of MUs.

On the other hand, Drinkwater et al. (2005) studied the effects of RT to failure on upper limbs strength of 26 male elite junior basketball players. All subjects had experience in weight-training. Muscular strength was evaluated after two different protocols of equated volume: repetitions to failure (four sets of six repetitions) and non-failure (8 sets of 3 repetitions), with similar intensities (~85–105% 1-RM). Rest interval differed between protocols, with 260 s between sets for repetitions to failure and 113 s for non-failure. Fatigue extent was measured before and after RT while muscle activation was not assessed. Results showed greater fatigue and increase in strength (almost twofold) for the failure group. These results suggest that fatigue caused by muscular failure may be related to greater muscle activation, which would explain the greater increases in muscle strength when performing repetitions to failure, at least for strength trained individuals. In fact, a recent study by Looney et al. (2015) seems to support such hypothesis. Employing a randomized within-subject design with 10 strength trained men, the authors investigated muscle activation at different intensities performed to submaximal and maximal repetitions (i.e., failure), comparing single set and drop set protocols. Even though fatigue levels were not assessed, results demonstrated greater EMG amplitudes whenever repetitions were performed to failure compared to submaximal repetitions of same intensity. Such findings suggest that, for strength trained individuals, HI-RT to muscle failure is necessary for maximal muscle activation, which may be related to the greater increases in muscular strength.

The lack of measurements of both fatigue levels and muscle activation in studies that compare HI-RT to failure or no failure hampers comprehension on how these mechanisms contribute to RT-related chronic adaptations. Additionally, no studies have directly compared hypertrophic responses after exercising to failure or no failure. The differences in subjects training status represent another problem, hindering the ability to draw relevant comparisons between studies. As the above studies seem to point, fatigue influence on muscle activation and adaptations can differ greatly depending on the subject training level. Apparently, for individuals untrained in strength, RT to failure is unnecessary for maximizing increases in muscle strength and muscle mass. Conversely, for trained individuals, repetitions to failure might result in increased muscle activation, which could explain the greater increases in muscle strength after protocols performed to muscular failure. However, it remains unclear if this fatigue-induced muscle activation truly reflects chronic increases in strength and muscle hypertrophy, and how differently it affects untrained and trained individuals.

## Muscular failure in low intensity resistance training

Recent studies have pointed muscular failure to be an important factor in order to maximize adaptations when RT is done at low intensities (LI-RT). However, no attempts have been made to directly compare the effects of training to muscle failure versus no failure on neuromuscular adaptations. As consequence, the researchers compare LI-RT with HI-RT, assuming the last as the ideal situation for increasing both muscle mass and strength.

Regarding untrained individuals, Mitchell et al. (2012) sought to determine whether different intensities would result in different chronic adaptations. In their study, 18 recreationally active men, with no weightlifting experience in the last year, performed 10 weeks of unilateral knee extension to failure. Each leg was randomly assigned in a counterbalanced way to one of the three possible unilateral training conditions: LI-RT (3 series at 30% 1-RM), HI-RT (3 sets at 80% 1-RM), and HI-RT with a single set. Results evidenced similar increases in isometric strength and muscle hypertrophy between protocols after 10 weeks. Despite no assessment on muscle activation was performed, it is possible that these adaptations are related to a similar MU recruitment between HI-RT and LI-RT protocols when both are performed to muscular failure, with no apparent advantage in performing HI-RT to failure, at least for untrained individuals.

On the other hand, Holm et al. (2008) investigated the effects of LI-RT and HI-RT on 11 sedentary young men. Participants performed five sets of unilateral knee extension for 12 weeks, training one leg with 70% 1-RM (eight repetitions) and the opposite leg with 15.5% 1-RM (1 repetition every 5th second for a total of 36 repetitions) in a random and counterbalanced way. Results demonstrated marked increases in quadriceps cross sectional area (three-fold) for HI-RT when compared to LI-RT. Muscle fatigue and activation levels were not assessed, but considering the rest period allowed between each contraction in LI-RT condition, it is possible to speculate that MUs recruitment would be too low compared to HI-RT due the lower fatigue level resulting from this protocol.

When it comes to strength trained individuals, results are more consistent. To study the influence of training intensity on muscle activation Schoenfeld et al. (2014) employed a within-subject design in which resistance-trained young men performed two protocols: high-load (75% 1-RM to failure); and low-load (30% 1-RM to failure). Participants performed the protocols in a counterbalanced way. Results showed greater peak and mean EMG activity during high-load, indicating that training to failure at low intensities does not result in maximal MUs activation for trained individuals. Conversely, another study by the same research group assessed muscle strength and mass in 24 men, all experienced in weight lifting, after 8 weeks (24 training sessions) of RT (Schoenfeld et al., 2015). Subjects were randomly assigned to one of two possible training conditions performed to failure: low-load (25–35 repetitions ~30–50% 1-RM) and high-load (8–12 repetitions at ~70–80% 1-RM). The outcomes demonstrate similar increases in muscle mass between the two training regimen. However, results evidenced greater improvements in strength for the high-load group. Results indicate that, even though LI-RT to failure is able to achieve hypertrophic levels similar to HI-RT, training at high intensities is necessary to maximize strength adaptations.

Considering the evidence to date, it seems plausible to assume that fatigue differently affects muscle adaptations when LI-RT is performed to muscular failure by different populations (trained and untrained). For untrained subjects, it is possible that protocols resulting in low fatigue, like the one used by Holm et al. (2008), are unable to maximally recruit high threshold MUs. Results could have been different if the low intensity repetitions were carried out near muscular failure, as in Sundstrup et al. (2012) study. For trained individuals, it is possible that, due RT-related neural adaptations, fatigue following LI-RT protocols would not be sufficient to maximally recruit the high threshold MUs of strength trained individuals. Thus, the similar hypertrophy between LI-RT and HI-RT would result from the greater time under tension when performing LI-RT to failure, which would maximally stimulate type I muscle fibers, promoting greater hypertrophic response.

Finally, some studies suggest that RT to failure for a prolonged period may result in overtraining; higher risk of musculoskeletal injury by repetitive effort; and stronger hemodynamic responses, with pressure peaks near muscular failure (MacDougall et al., 1985; Stone et al., 1996). Therefore, guidelines recommend performing RT to a level of substantial fatigue (i.e., submaximal efforts), ensuring strength and muscle mass increases, while avoiding failure (Pollock et al., 2000; Mazzeo and Tanaka, 2001; Haskell et al., 2007). These findings can be especially important to populations that may have adverse effects when performing HI-RT (e.g., elderly and hypertensive), where performing LI-RT with level of substantial fatigue can be a viable alternative for maximize strength gains and muscle mass.

## Conclusion

In conclusion, considering the evidence regarding untrained subjects, it seems plausible to suggest that HI-RT to failure is not necessary for maximal increases in strength and hypertrophy. On the other hand, repetitions to failure seem essential for increases in muscle strength and mass of similar magnitude to HI-RT when performing LI-RT. When it comes to trained individuals, evidence show greater increases in muscle strength after HI-RT performed to muscle failure compared to no failure. Similarly to untrained individuals, muscle failure at LI-RT might be an interesting strategy for maximizing muscle hypertrophy. However, it does not promote maximal increases on muscle strength when performed by strength trained individuals.

Future studies should be conducted to determine how fatigue extent influences MUs recruitment and RT-related muscle adaptations on strength trained and untrained individuals.

## Funding

This publication was supported by São Paulo Research Foundation (FAPESP) grants #2016/00314-3.

### Conflict of interest statement

The authors declare that the research was conducted in the absence of any commercial or financial relationships that could be construed as a potential conflict of interest.
